# Psychological distress and trust in healthcare: individual perceptions and national contexts - a multilevel analysis

**DOI:** 10.1186/s12913-026-14948-7

**Published:** 2026-06-18

**Authors:** Juliane Heise

**Affiliations:** University of Technology Chemnitz, Chemnitz, Germany

**Keywords:** Trust in doctors, Healthcare fairness, Access inequality, Distress, Multilevel analysis, Income inequality, Human development index (HDI)

## Abstract

**Aim:**

This study examines the association between trust in doctors, income-based perceived fairness, income-based access inequality in healthcare, and self-reported psychological distress, considering individual perceptions and country level socioeconomic context.

**Methods:**

ISSP 2021 Health and Health Care data were analyzed (*N* = 26 023; 21 countries). A multilevel logistic regression model was estimated. Country level indicators included income inequality (Gini coefficient), Human development index (HDI) and average confidence in the healthcare system.

**Results:**

Compared with neutral trust, trusting doctors was associated with lower odds of distress (OR = 0.78, 95% CI 0.72–0.84), while distrust was associated with higher odds (OR = 1.14, 95% CI 1.01–1.30). Strong perceptions of healthcare injustice were associated with elevated distress, reflected in higher odds among those rating the system as very unfair (OR = 1.26 95% CI 1.16–1.37) and those perceiving access as harder for the poor (OR = 1.30 95% CI 1.20–1.41). At the country level, higher income inequality (OR = 1.26, 95% CI 1.10–1.44) was associated with higher distress, whereas HDI and average confidence in the healthcare system was not significantly related to distress.

**Discussion:**

Overall, the findings highlight that psychological distress is closely linked to both individual-level institutional perceptions and broader societal inequalities, with trust in doctors emerging as a particularly strong psychosocial resource.

**Supplementary Information:**

The online version contains supplementary material available at 10.1186/s12913-026-14948-7.

## Introduction

Psychological distress refers to a state of recurrent emotional suffering that is closely linked to psychological strain, such as stress, worry, and feelings of unhappiness or depressed mood [69]. These manifestations of distress are highly prevalent in the general population and constitute an important public health concern, as they are associated with impaired well-being and reduced functioning in everyday life [23]. In public health research, psychological distress is widely conceptualized as an indicator of individuals’ subjective mental health burden and their emotional responses to social and psychological stressors encountered in daily life [48]. Psychological distress cannot be understood solely as an individual phenomenon but emerges from the interaction between personal circumstances and broader social and institutional contexts that structure access to resources, shape expectations, and influence perceptions of fairness and security [6, 31, 33, 52].

Within this institutional context, trust, fairness, and access to healthcare are closely intertwined and jointly shape how individuals experience health-related uncertainty. Institutional trust refers to individuals’ confidence that healthcare institutions and professionals act competently, reliably, and in accordance with shared norms [67]. Trust is particularly salient in healthcare, as medical encounters are characterized by uncertainty, dependency, and information asymmetries. When trust is high, individuals are more likely to feel secure and interpret both bodily and emotional symptoms as manageable, whereas low trust may foster hypervigilance, anxiety and uncertainty that contribute to symptoms of psychological distress [2, 3, 26]. Accordingly, trust in doctors has been described as a key psychosocial resource supporting care-seeking, treatment adherence, and recovery [53]. In addition to experiences with individual providers, people also evaluate the healthcare system more broadly. When healthcare systems are perceived as unfair, individuals may experience feelings of exclusion, low social status, and lack of control which are factors known to increase psychological strain and distress [22, 24, 49]. From social and organizational justice perspectives, fairness encompasses both distributive aspects, such as equitable access social groups, and procedural aspects, including respectful treatment, transparency, and voice in medical encounters [32, 43]. Closely related to trust and fairness, perceptions of income-stratified access to healthcare may undermine feelings of institutional reliability and security, even among individuals who are not personally disadvantaged. By signaling structural inequality, such perceptions can generate psychological distress through concerns about future vulnerability or exclusion from adequate care, as perceived inequalities act as psychosocial stressors that erode social cohesion and institutional trust [14, 36]. Taken together, trust in healthcare providers, perceived access inequality, and perceived fairness shape whether healthcare alleviates or increases psychological distress.

At the macro level, national contexts provide broader structural conditions for distress-related health outcomes. More unequal societies tend to exhibit stronger social stratification, weaker social cohesion, and heightened status competition, which have been linked to chronic psychosocial stress and poorer health outcomes [9, 62]. From a psychological inequality perspective, stress arises not only from absolute deprivation but also from relative disadvantage, social comparison, and perceived unfairness, contributing to psychological distress across the population [37]. Importantly, prior research suggests that the psychological effects of inequality are not the same for everyone. In more unequal societies, individual well-being depends more strongly on relative income and subjective social status, meaning that social position becomes more consequential under conditions of greater inequality [11, 50]. Consistent with this, inequality is linked more strongly to poorer psychological health among people facing financial strain and tends to widen existing socioeconomic disparities in depression risk [42, 54]. This suggests that macroeconomic conditions may be especially consequential for individuals in structurally disadvantaged positions [47, 61]. Beyond inequality, national economic development is commonly theorized as a protective factor for health, as it expands the material capacity of health and welfare systems, improves living standards, and reduces economic insecurity [8, 10, 27, 56]. A large body of research in public health and economics shows that higher levels of economic development are consistently associated with lower mortality and higher life expectancy, partly because rising incomes increase both individual demand for health-relevant goods and services and public investment in health infrastructure and provision [16, 27, 28, 55]. However evidence suggests that the health benefits of economic growth depend on governance quality and the distribution of resources, indicating that growth alone is not sufficient to improve population health [18, 27]. Wealthier societies are generally better positioned to provide universal healthcare coverage and social protection, potentially buffering health-related uncertainty and stress. At the same time, high-income societies may also generate new stressors through intensified competition, demanding work environments, and urbanization [17, 25]. Thus, psychological distress is shaped by individual and institutional experiences. At the macro level, healthcare systems differ as national contexts that influence collective expectations about care and security during illness [38]. Institutional theory further suggests that macro-level trust climates interact with individual experiences. In contexts of high institutional trust, personal trust in doctors may be more easily sustained and translated into feelings of security, whereas fragile institutional environments may generate stress even among individuals who personally trust their physician [41].

Although previous research has examined psychological distress, trust in doctors, perceived fairness of the healthcare system, and perceived inequalities in healthcare access, these factors have often been studied separately, leaving several important gaps. Much of the existing literature focuses on single-country settings, clinical or otherwise selective samples, or narrowly defined mental health outcomes, whereas less is known about how healthcare-related trust and fairness perceptions relate to general psychological distress in the broader population across countries [2, 26, 68]. Moreover, although institutional trust is widely acknowledged as a relevant determinant of health, its relationship with stress-related psychological complaints remains underexplored across diverse national and healthcare system contexts [20, 45].

Against this background, the present study makes three contributions. First, it examines psychological distress as a broad, population-relevant indicator of mental health burden rather than focusing on a specific clinical disorder. Second, it draws on data from the International Social Survey Programme (ISSP), which provides a large cross-national, population-based, and harmonized survey framework, thereby enabling the analysis of healthcare-related perceptions in a comparative setting using broadly comparable measures across countries. This is an important advantage over studies limited to single-country or more selective samples. Third, the study investigates whether trust in doctors, perceived fairness of the healthcare, and perceived inequality in healthcare access are associated with psychological distress across countries, while also linking these individual-level perceptions to country-level indicators of income inequality, socioeconomic development and average confidence in the healthcare system. In doing so, it moves beyond single-country and single-level approaches and identifies system-related correlates of distress that are directly relevant to public health and health service provision.

This study addresses those research gaps by posing two central research questions:

(1) Are psychological distress levels associated with individual perceptions of trust, fairness, and healthcare access across countries? (2) Do country-level income inequality, socioeconomic development, and average confidence in the (country-specific) healthcare system explain variation in psychological distress?

**Fig. 1:**
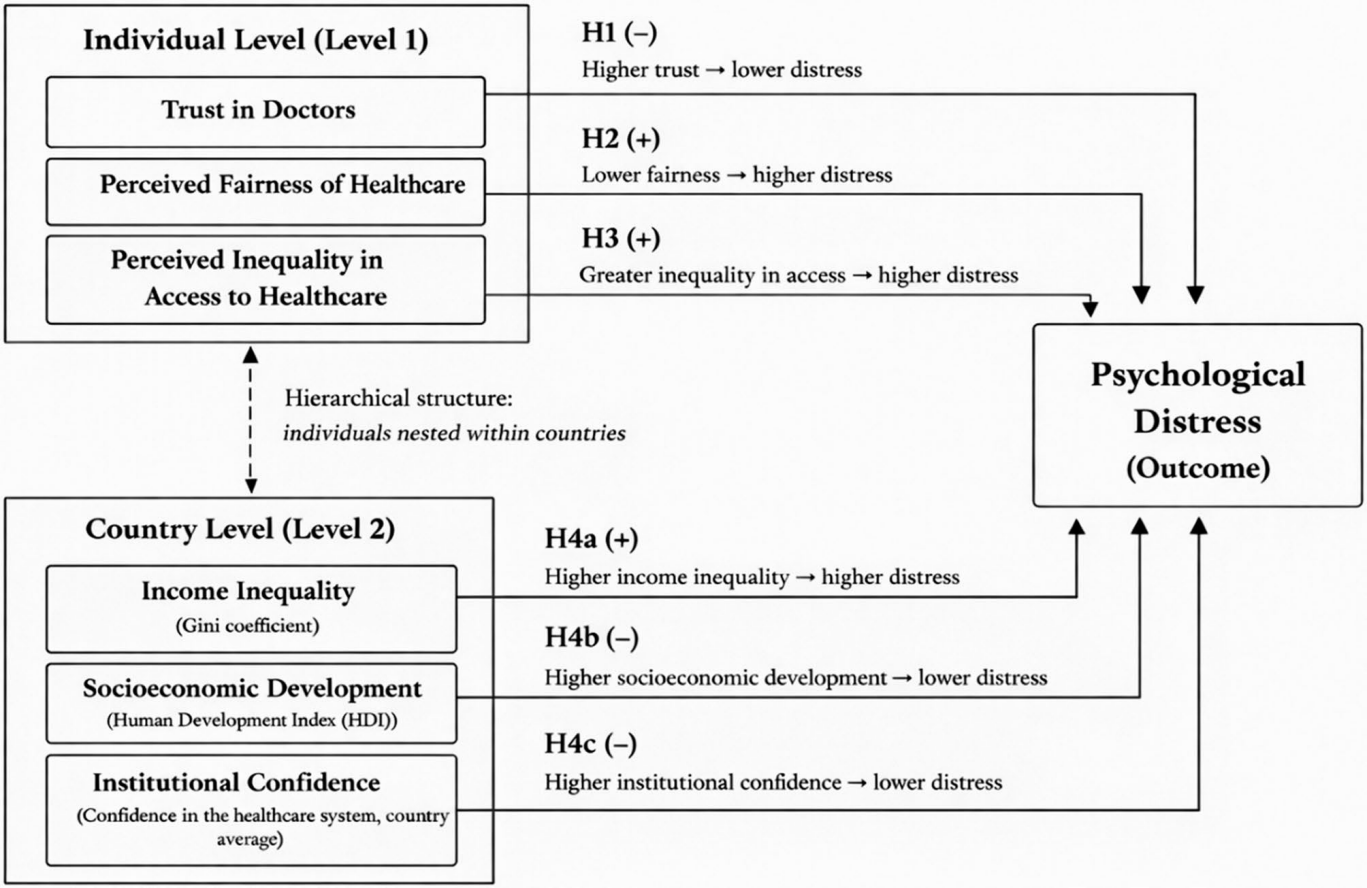
Conceptual framework of country- and individual-level determinants of psychological distress

Based on this framework, the following hypotheses guide the analysis (see also Fig. 1):

### H1 (Trust–Distress)

Individuals with higher trust in doctors have lower odds of experiencing psychological distress.

### H2 (Fairness–Distress)

Individuals who perceive healthcare as low in fairness report higher psychological distress.

### H3 (Access Inequality-Distress)

Perceived inequality in access to healthcare is associated with higher psychological distress.

Country Level:

### H4a (Income Inequality)

Higher income inequality at the country level is associated with higher levels of psychological distress.

### H4b (Socioeconomic development)

Higher national socioeconomic development, is associated with lower levels of psychological distress.

### H4c (Institutional Confidence)

Higher average confidence in the healthcare system at the country level is associated with lower levels of psychological distress.

The relevance of these hypotheses becomes particularly evident from a public health and health services perspective. Psychological distress is a key population-level indicator, as it is linked to adverse outcomes including increased mortality risk as well as higher healthcare utilisation and costs [12, 44]. In addition to clinical efficacy, health systems are assessed based on their intrinsic performance goals, which include responsiveness to people’s reasonable expectations (such as autonomy, dignity, and communication) and equity in the financing and accessibility of care [66]. Because it influences whether people seek care, disclose information, follow advice, and take part in preventive programs, trust in medical professionals and the healthcare system is a public health resource [7]. Conversely, low trust is associated with delays in care and psychological distress. Similarly, both perceived unfairness and access are important for public health because they serve as psychosocial stressors and may indicate institutional exclusion and unreliability [22]. A cross-national approach is essential because trust climates and inequality contexts differ systematically across countries, enabling health system learning about which institutional conditions are linked to population mental health and where equity-oriented reforms may yield the greatest benefit [66].

Taken together, these considerations demonstrate that trust, fairness, and access are not merely individual perceptions but modifiable features of healthcare systems that can influence population mental health and the effectiveness of health service provision.

## Methods

The analysis uses data from the International Social Survey Programme (ISSP) 2021 module on Health and Health Care [21]. The ISSP is an international collaborative survey programme that provides harmonized cross-national data on social attitudes and experiences. The integrated ISSP 2021 Health and Health Care II data file includes nationally representative adult samples from multiple countries across different world regions, enabling cross-national analysis of attitudes and experiences of health and healthcare. Respondents were sampled using probability-based methods within each country, and the survey was administered in face-to-face or mixed modes depending on national procedures. The main analysis was based on a final analytical sample of 26,023 individuals nested within 21 countries. Although the ISSP dataset originally included 30 countries, the restriction was motivated by concerns about potential confounding from unobserved macro-level characteristics, such as political system stability, institutional structures, and cultural differences, that are difficult to adequately capture with the available data but may systematically differ between high- and low-income countries. To enhance comparability across countries and reduce the risk of such confounding, the final analytical sample primarily includes high-income and middle-income countries with more similar institutional and socio-economic frameworks. As a robustness check, the analysis was additionally conducted using an extended country sample; these results are reported in the Supplementary Material Table S5 (including India, Mexico, the Philippines, Russia, South Africa, Suriname and Thailand; Taiwan and China were still excluded because of missing and insufficiently reliable macro-level/control data).

Listwise deletion was applied as the proportion of missing values for each variable remained below the critical threshold of 5% [40].

### Operationalization

Psychological distress was measured using the item (*Q10: During the past 4 weeks*,* how often…Q16c:…have you felt unhappy and depressed?*). It was originally measured on a five-point Likert scale ranging from (1) *never* to (5) *very often*. For the analyses, the original scale was dichotomized into low distress (1–2) *never/seldom*) and elevated distress (3–5) *sometimes* to *very often*. This coding strategy emphasizes the distinction between respondents with no or infrequent distress and those experiencing at least occasional symptoms, thereby facilitating a more straightforward interpretation in terms of the presence versus absence of meaningful psychological distress. Although dichotomization reduces variation in the original measure and may entail some loss of information, it can be useful when the analytical focus is on identifying individuals with elevated levels of distress rather than modeling fine-grained differences across response categories [5, 35].

#### Individual-level predictors (level 1)

Trust in doctors was measured using a survey item assessing general confidence in medical professionals (*Q10: How much do you agree or disagree with the following statements about doctors in general in [country]? Q10a: All things considered doctors can be trusted*). Responses were recorded on a five-point Likert scale ranging from (1) *strongly agree* to (5) *strongly disagree*. Although the item refers specifically to doctors as a professional group and is therefore interpreted as a measure of domain-specific trust, it may partly reflect broader attitudes toward the healthcare system. The results should therefore not be understood as referring exclusively to interpersonal trust in individual physicians.

Perceived fairness of healthcare (income-based) was assessed using an item evaluating whether it is considered fair or unfair that people with higher incomes can afford better healthcare than those with lower incomes (Q3: *Is it fair or unfair that people with higher incomes can afford better health care than people with lower incomes?*). Responses were measured on a five-point Likert scale from (1) *very fair* to (5) *very unfair*.

Perceived access inequality in healthcare (income-based) was measured by an item assessing whether access to healthcare is perceived as easier or harder for rich people compared to poor people in the respondent’s country (*Q7: In [country]*,* do you think it is easier or harder to get access to health care …Q7a: … for rich people than for poor people?*). Responses were measured on a five-point Likert-type scale ranging from (1) *much easier for rich people* (5) *to much harder for rich people*.

#### Country-level variables (level 2)

Three country-level variables were used to capture broader institutional and socioeconomic contexts. The first macro-level indicator captures income inequality at the country level, measured using the Gini coefficient. The Gini coefficient is a widely used summary measure of income distribution that ranges from 0 (perfect equality) to 100 (perfect inequality), indicating the extent to which the distribution of income among individuals or households deviates from a perfectly equal distribution [34]. The Gini coefficients for this analysis were obtained from the World Bank World Development Indicators, based on household survey data. Data for 2021 were used wherever available; otherwise, the most recent available year was selected (Australia: 2020; Germany: 2020; Iceland: 2019; Japan: 2020) [63]. As the underlying welfare concept in the World Bank data may refer to either income or consumption depending on country-specific data availability, some cross-national differences in measurement should be noted. For New Zealand, the Gini coefficient was obtained from the World Income Inequality Database (United Nations University World Institute for Development, [60]). To ensure the highest possible comparability between data sources, the New Zealand value was cross-checked against other available estimates. 

The second macro-level indicator captures the level of socioeconomic development at the country level, measured using the Human Development Index (HDI). The HDI is a composite index that combines three key dimensions of human development: life expectancy at birth (as an indicator of health), educational attainment (measured by mean and expected years of schooling), and gross national income per capita (as a proxy for standard of living) (United Nations Development Programme [UNDP], [59]). The index ranges from 0 to 1, with higher values indicating higher levels of human development. Compared to purely economic indicators such as GDP per capita, the HDI provides a broader and more comprehensive measure of development by incorporating both social and economic dimensions of human development [13, 19, 46]. This multidimensional approach reflects the understanding that economic output alone does not adequately capture other central dimensions of human development, such as health and education. This makes it particularly suitable as a macro-level indicator in cross-national research, as it captures structural differences in living conditions, capabilities, and opportunities that may influence individual outcomes. HDI values for this analysis were obtained from the United Nations Development Programme Human Development Data Center [59]. Data for the year 2021 was used for all countries to ensure comparability across cases.

The third macro-level indicator reflects the average level of confidence in the healthcare system within each country. This measure was constructed by aggregating individual survey responses on confidence in the healthcare system to the national level, resulting in a country mean that represents the overall trust in the healthcare system. Respondents rated their confidence on a 5-point scale ranging from (1) *complete confidence* to (5) *no confidence* at all.

The observed ranges of all three Country-Level indicators in the present sample are reported in the Supplementary Material (Table S1). To facilitate comparability across countries, all Country-Level indicators were standardized.

The model adjusts for sex (male/female), age (mean-centred, ≤ 80 years), educational attainment (primary or less, secondary, tertiary), place of residence (rural to big city), and self-rated health (poor/fair, good, very good, excellent).

### Statistical analysis

A logistic generalized linear mixed model (GLMM) was employed. Conventional significance thresholds (***p < .001, **p < .01, *p < .05) were used to interpret the results. The intraclass correlation coefficient (ICC) derived from this model indicated that approximately 3.4% of the variance in distress was attributable to between-country differences, justifying the use of multilevel modelling. Subsequently, a Level-1 model including individual-level predictors and a Level-2 model incorporating country-level predictors were estimated. The Level-2 model demonstrated the best overall model fit examined via the Akaike Information Criterion (AIC) and Bayesian Information Criterion (BIC). No multicollinearity issues were detected among the predictors (all variance inflation factors were within acceptable range (1.02–1.56)). The random intercept model was favored over a fixed-effects-only model based on a likelihood ratio test, indicating between-country variance in distress (as also evidenced by the estimated random intercept variance).

All analyses were conducted using R (version 4.4.3) and the lme4 package for multilevel modelling.

## Results

An overview of the descriptive statistics is provided in Tables 1 and 2; further details for the country variables (incl. Gini coefficient, HDI, Average confidence in the healthcare system) are reported in Table S1 in the supplementary material. Approximately two thirds of the sample reported low levels of psychological distress: 66.9% indicated that they felt unhappy or depressed never or seldom during the past four weeks, whereas 33.1% reported experiencing these feelings sometimes to very often, indicating elevated psychological distress.

**Table 1:** Characteristics of the sample population (categorical variables)

Variables	*N*	percentages (%)
Distress level Never/Seldom Sometimes-Very often	17 399 8 624	66.9 33.1
Doctors can be trusted Strongly Agree Agree Neither agree nor disagree Disagree/ strongly disagree	3 878 16 014 4 271 1 860	14.9 61.5 16.4 7.1
Fairness of healthcare (income-based) Very fair Somewhat fair Neither fair nor unfair Somewhat unfair Very unfair	1 297 3 639 5 197 7 265 8 625	5.0 14.0 20.0 27.9 33.1
Access inequality (income-based) Much easier (rich) Somewhat easier (rich) About the same Somewhat harder/ Much harder (rich)	12 397 7 842 5 027 757	47.6 30.1 18.1 2.9
Sex male female	12 040 13 983	46.3 53.7
Education level Primary education or less Secondary education Tertiary education	750 14 223 11 050	2.9 54.7 42.5
Place of residence Farm/ home in country Country village Town or small city Suburb of a big city Big city	1 014 7 315 7 377 4 171 6 146	3.9 28.1 28.3 16.0 23.6
Self-rated health Poor/ fair Good Very good Excellent	6 010 10 691 7 538 1 784	23.1 41.1 29.0 6.9

Notes: *N* = 26 023 (Data source: ISSP2021, own presentation)

Overall levels of trust in doctors were high. A clear majority of respondents expressed positive trust evaluations: 61.5% reported that they agreed and 14.9% that they strongly agreed that doctors can be trusted. For perceived fairness of healthcare (income-based) most respondents expressed critical views. While 19% of respondents considered income-related differences in healthcare fair or somewhat fair, a majority perceived them as unfair, with over half rating such inequalities as somewhat or very unfair. Perceived access inequality between rich and poor was pronounced. 77.7% respondents reported that access to healthcare is at least somewhat easier for rich people, whereas only 18.1% perceived access as roughly equal, and only a negligible share believed it to be harder for the rich.

**Table 2:** Characteristics of the sample population (metric variables)

Variables	mean	SD	Min	Max
Gini coefficient HDI	30.6 0,924	4.2 0,035	24.1 0,852	39.7 0,969
Average confidence in the healthcare system	3.44	0.33	2.75	3.93
Age (in years)	48.3	16.47	16	80

Notes: *N* = 26 023 (Data source: ISSP 2021, own presentation)

Income inequality varied considerably across countries, with more egalitarian income distributions in countries such as Slovakia and substantially higher inequality in countries such as the United States. Human development also differed markedly across countries, with comparatively lower levels in countries such as Hungary and higher levels in countries such as Norway. Average confidence in the healthcare system showed less extreme variation but still differed across countries, with relatively lower levels in Poland and higher levels in countries such as Norway.

**Fig. 2:**
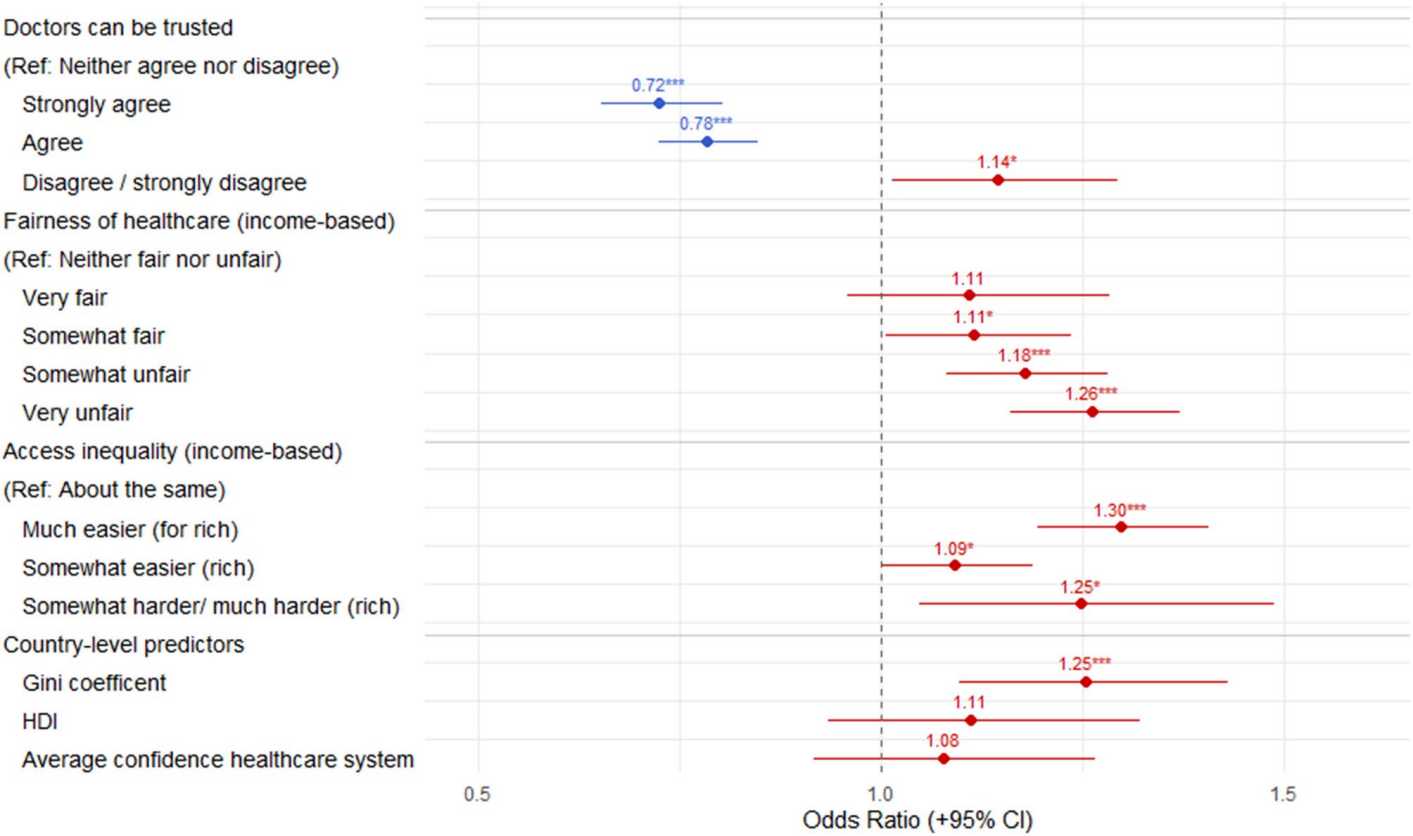
Logistic multilevel model including the variables of the hypotheses. Notes: Dependent variable: psychological distress (Ref = never/seldom). The model was estimated including control variables; for reasons of clarity, only the hypotheses-related variables are displayed (for full model see supplementary material Table S4). Points indicate odds ratios (OR), horizontal bars represent 95% confidence intervals. Statistical significance is indicated as ***p < .001, **p < .01, *p < .05

Figure 2 displays the results of the hypotheses variables derived from the multilevel logistic regression model estimating the odds of psychological distress. The results of the complete model are shown in Table S2 in the supplementary material.

Trust in doctors was significantly associated with psychological distress when compared to a neutral level of trust. Relative to individuals who neither agreed nor disagreed that they trust doctors, those who reported strongly agreeing had significantly lower odds of distress (OR = 0.72, 95% CI 0.65–0.80, *p* < .001), as did those who agreed (OR = 0.78, 95% CI 0.72–0.84, *p* < .001). In contrast, respondents who disagreed or strongly disagreed that they trust doctors exhibited higher odds of psychological distress compared to the neutral group (OR = 1.14, 95% CI 1.01–1.30, *p* = .030).

Perceived fairness of healthcare was associated with psychological distress across multiple levels of evaluation. Compared to respondents who perceived such differences as neither fair nor unfair, those who rated them as very unfair (OR = 1.26, 95% CI 1.16–1.37, *p* < .001) and somewhat unfair (OR = 1.18, 95% CI 1.08–1.28, *p* < .001) had significantly higher odds of distress. In addition, those who perceived such differences as somewhat fair also showed slightly higher odds of distress (OR = 1.11, 95% CI 1.00–1.23, *p* = .040), whereas no statistically significant difference was observed for those who rated the system as very fair (OR = 1.11, 95% CI 0.96–1.28, *p* = .169).

Perceived access inequality was also related to psychological distress. Compared to individuals who perceived access as about the same for rich and poor people, those who reported that access was somewhat or much harder for rich people showed higher odds of distress (OR = 1.25, 95% CI 1.05–1.50, *p* = .013). Similarly, perceiving access as much easier for rich was associated with increased odds of distress (OR = 1.30, 95% CI 1.20–1.41, *p* < .001), and perceiving access as somewhat easier for rich people was also associated with slightly higher odds (OR = 1.09, 95% CI 1.00–1.19, *p* = .048).

At the country level, income inequality was positively associated with psychological distress. Higher values on the Gini coefficient were linked to higher odds of distress (OR = 1.26, 95% CI 1.10–1.44, p = < 0.001). In contrast, HDI and average country-level confidence in the healthcare system were not significantly associated with psychological distress.

Several control variables were significantly associated with psychological distress. Women had higher odds of psychological distress than men (OR = 1.47, 95% CI 1.39–1.56, *p* < .001), while age was associated with lower odds of distress (OR = 0.98, 95% CI 0.98–0.98, *p* < .001). Higher levels of education were also associated with reduced odds of distress (secondary: OR = 0.82, 95% CI 0.69–0.97, *p* = .024; tertiary: OR = 0.72, 95% CI 0.61–0.86, *p* < .001). Self-rated health showed the strongest associations, with substantially lower odds of psychological distress among respondents reporting good (OR = 0.32, 95% CI 0.30–0.34, *p* < .001), very good (OR = 0.15, 95% CI 0.14–0.16, *p* < .001), or excellent health (OR = 0.08, 95% CI 0.07–0.09, *p* < .001). Place of residence was also associated with psychological distress, with higher odds observed across all non-rural categories compared to rural areas, including country villages (OR = 1.29, 95% CI 1.10–1.53, *p* = .002), towns or small cities (OR = 1.34, 95% CI 1.15–1.58, *p* < .001), suburbs of big cities (OR = 1.30, 95% CI 1.10–1.54, *p* = .002), and big cities (OR = 1.39, 95% CI 1.18–1.63, *p* < .001).

Results from the extended country sample were largely consistent with the main findings (see Table S3 in the supplementary material). In particular, the association between trust in doctors and psychological distress remained stable (e.g., strongly agree: OR = 0.71, 95% CI 0.65, 0.77, *p* < .001 vs. 0.72 95% CI 0.65, 0.80 in the restricted sample). Some differences emerged for healthcare system perceptions: associations for perceived fairness were attenuated and partly lost statistical significance, whereas effects of perceived access inequality became stronger (e.g., OR = 1.51, 95% CI 1.33, 1.72, *p* < .001 for “somewhat harder/much harder for rich people”). At the country level, HDI showed a significant positive association in the extended sample (OR = 1.31, 95% CI 1.09, 1.57, *p* = .004).

## Discussion

This study examined how trust in doctors, perceived fairness of the healthcare system, and perceived inequalities in healthcare access relate to psychological distress using a multilevel framework that incorporates country-level indicators. Several important findings emerged, highlighting the relevance of both individual-level institutional perceptions and broader social contexts for psychological health. First, trust in doctors showed a strong and graded association with psychological distress. Compared to individuals with a neutral level of trust, those who expressed agreement or strong agreement that they trust doctors had substantially lower odds of distress, whereas those who disagreed exhibited higher odds. This clear dose–response pattern suggests that trust in physicians functions as a psychosocial resource that buffers stress-related health complaints. These mechanisms align with sociological and psychological theories of trust. Prior research highlights trust as central for managing vulnerability in clinical encounters, particularly through relationship features such as communication quality and perceived competence [29, 57]. This present study extends previous research by demonstrating this association in large, general population samples across multiple countries and by linking trust in doctors specifically to self-reported distress. Psychological symptoms often represent embodied stress responses, and high trust may attenuate these responses by reducing perceived threat and enhancing feelings of control and support [26]. The results show a graded association across levels of trust. Agreement and strong agreement with trusting doctors are linked to lower levels of distress, whereas disagreement is linked to higher levels of distress. This suggests that a lack of trust is not neutral but may itself act as a source of stress.

Second, the findings regarding perceived fairness of income-based differences in healthcare access partly support the hypothesis but also refine it. Respondents who perceived the system as somewhat unfair or very unfair exhibited significantly higher odds of psychological distress compared to the neutral reference group, while even those who perceived the system as somewhat fair showed a small but statistically significant increase. This indicates a graded (dose–response) association rather than a strict threshold effect: psychological distress increases progressively with more negative (and even slightly positive) deviations from neutrality, instead of rising only at the extreme end of perceived injustice. This pattern aligns with stress and justice theories, which emphasize that chronic exposure to severe or highly salient injustice is more harmful to health than minor or occasional complaints [15, 29]. But it also suggests that even moderate or less salient perceptions of unfairness may already be sufficient to affect psychological well-being. Individuals who view the healthcare system as fundamentally unfair may anticipate mistreatment, exclusion, or neglect. Such expectations can increase vigilance and psychological stress even in the absence of direct personal harm. The results therefore suggest that strong perceptions of institutional injustice can affect health through stress-related pathways, while the observed gradient indicates that these mechanisms may operate along a continuum rather than only under conditions of extreme perceived injustice. In the full country sample, including low-income countries, only somewhat unfair and very unfair perceptions were significantly associated with psychological distress, suggesting that the relationship is mainly driven by clearly negative fairness evaluations across the broader set of country contexts.

Third, in contrast to general fairness perceptions, perceived inequality in healthcare access showed a clearer and more consistent association with distress. Individuals who perceived access to healthcare as much easier for rich people reported substantially higher levels of distress compared to those who perceived access as roughly equal. Importantly, perceiving access as somewhat harder/ much harder for the rich was also associated with higher distress, indicating that both extremes of pronounced access inequality point in the same direction. This suggests that strongly perceived access inequality itself is stress-inducing, regardless of whom it favors. Previous research has shown that perceived social and institutional inequalities can function as psychosocial stressors by undermining feelings of security, trust, and social cohesion, even among individuals who are not personally disadvantaged [14, 30]. In the context of healthcare, awareness that access is strongly stratified by socioeconomic status may heighten concerns about vulnerability, exclusion, or future need for care, thereby increasing distress [4, 36]. The absence of an association for more moderate access differences further suggests a non-linear relationship, in which only clearly perceived and extreme forms of access inequality are psychologically consequential.

However, the observed associations should also be interpreted considering potential reverse or reciprocal processes. Psychological distress may shape how individuals evaluate social and institutional actors. Individuals experiencing distress may hold more negative expectations about the trustworthiness of others and may therefore be more likely to report lower trust in doctors or to perceive healthcare arrangements as unfair [51]. Thus, the findings may partly reflect distress-related negative expectations rather than only the effect of healthcare-related perceptions on distress.

At the macro level, the findings of this study further nuance the role of context. Country-level income inequality, as measured by the Gini coefficient, was significantly associated with higher distress. This finding supports theories of psychosocial inequality, which argue that unequal societies generate chronic stress through mechanisms such as social comparison, status anxiety, and perceived unfairness [30]. Importantly, this association persisted after adjustment for individual socioeconomic characteristics, suggesting that inequality exerts a contextual influence on health beyond personal circumstances. These results are consistent with previous evidence showing that income inequality is linked to worse mental and subjective health outcomes at the population level, even after accounting for individual resources [1, 4, 30, 58]. The findings therefore complement the individual-level access inequality results, indicating that both perceived and structural inequalities are relevant for distress.

In contrast to expectations, no significant association was observed between HDI and psychological distress in the present analysis. A likely explanation is the relative homogeneity of the included countries, as low- and lower-middle-income countries were excluded from the main analysis. This restriction reduced the variation in HDI across countries and may therefore have limited its explanatory power at the macro level. In this more homogeneous group of countries, psychological distress may be less strongly related to broad structural indicators such as HDI and more strongly shaped by psychosocial, cultural, or institutional factors that are not fully captured in the present analysis. By contrast, in the extended country sample, higher HDI was significantly associated with higher reported distress. This difference may partly reflect the broader HDI range in the extended country sample, which included substantially more lower to middle HDI countries and therefore provided greater variation for macro-level associations to emerge. However, this finding should be interpreted with caution because in such a diverse sample, macro-level associations may be confounded by country-level characteristics not accounted for in the present study, including differences in political systems, institutional stability, and cultural contexts. One possible explanation is that higher-HDI countries may have greater awareness of mental health issues and more developed systems of detection, diagnosis, and reporting, which can contribute to higher reported levels of distress without necessarily indicating worse underlying mental health [64, 65]. At the same time, more affluent societies may also expose individuals to specific forms of strain, including stronger performance pressures, more competitive social environments, and stress associated with demanding work and urban living [25].

Country-level confidence in the healthcare system was not significantly associated with distress once individual trust was accounted for. This suggests that personal perceptions and experiences may be more consequential for individual health than the general trust climate of a country. Similar conclusions have been drawn in previous research, which shows that individual trust in healthcare providers is more strongly related to health outcomes than aggregate measures of institutional trust [39].

Notably, the country-level factors included in the model accounted for only a small share of the variance in the outcome (3.4%), suggesting limited explanatory power at this level.

Several limitations should be acknowledged. The cross-sectional design precludes causal inference, and reciprocal relationships between psychological distress and perceptions of trust and fairness cannot be ruled out. Distress was measured using a single self-report item. Although this item captures a general subjective sense of unhappiness and distress, it does not reflect the multidimensional nature of psychological distress and may be more closely related to negative affects or depressive symptomatology than to the broader construct. In addition, the use of a single-item measure limits measurement precision and does not allow internal consistency reliability to be assessed. The findings should therefore be interpreted with appropriate caution. Furthermore, all key individual-level variables were self-reported and may be influenced by reporting styles or negative effect, potentially inflating some associations, although subjective perceptions are substantively relevant given the study’s focus. The measures of perceived fairness and access inequality were relatively broad and may not fully capture more specific experiences of institutional unfairness or unequal access to healthcare.

A further limitation concerns the relatively small number of level-2 units in the multilevel model. The final analytical sample included only 21 countries, which is below the number of level-2 units often recommended for estimating multilevel logistic regression models. This may affect the reliability and precision of the country-level estimates. Finally, some caution is warranted regarding the cross-national comparability of the Gini coefficient, as the underlying welfare concept in the World Bank data may refer to either income or consumption depending on the country. Future research could benefit from more detailed indicators of experienced inequality and from incorporating additional country-level characteristics, such as healthcare system features or social protection policies.

## Conclusion

This study aimed to examine two central questions: (1) whether psychological distress is associated with individual perceptions of trust, fairness, and access within healthcare systems, and (2) whether country-level factors such as income inequality, socioeconomic development, and national confidence in the healthcare system help explain cross-national variation in distress.

Psychological distress is closely linked to how individuals perceive healthcare institutions and the broader social context in which they are embedded. Trust in doctors emerges as a particularly important psychosocial resource, showing a clear protective association with distress, while perceptions of income-related unfairness and access inequality are associated with elevated distress. These findings underscore that institutional relationships are not merely background conditions but form part of the social environment through which stress becomes embodied. At the macro level, the results highlight that structural conditions matter alongside individual perceptions. Higher income inequality was consistently related to higher distress, suggesting that living in more unequal societies may generate psychosocial stress beyond individual socioeconomic position. However, no association was found between socioeconomic development and national-level confidence in the healthcare system and psychological distress, suggesting that macro-level institutional trust may operate differently than interpersonal trust in shaping stress-related outcomes. A significant association between socioeconomic development and psychological distress emerged only in the extended country sample included as a robustness check, indicating that this relationship may be sensitive to sample composition and country heterogeneity.

Taken together, the findings emphasize that psychological distress cannot be fully understood through individual characteristics alone. Instead, it reflects the interplay between personal experiences with healthcare institutions and wider societal contexts. The results suggest that trust in medical professionals and individuals’ perceptions of fairness and access within healthcare systems are closely associated with psychological distress. While causal conclusions cannot be drawn due to the cross-sectional design, these findings highlight the potential relevance of institutional experiences and broader social conditions for population mental health.

## Supplementary Information

Below is the link to the electronic supplementary material.

Supplementary Material 1

## Data Availability

The data used in this study are publicly available from the International Social Survey Programme (ISSP) Health and Health Care 2021 dataset and can be accessed via: https://www.gesis.org/en/issp/data-and-documentation/health-and-health-care/2021.

## Code availability

The code of this study is available from the corresponding author upon reasonable request.

## References

[1] Acheampong AO, Opoku EEO. Analyzing the health implications of rising income inequality: What does the data say? Econ Transition Institutional Change. 2024;32(4):1003–35. 10.1111/ecot.12410.

[2] Ahnquist J, Wamala SP, Lindström M. What has trust in the health-care system got to do with psychological distress? Analyses from the national Swedish survey of public health. Int J Qual Health Care. 2010;22(4):250–8. 10.1093/intqhc/mzq024. 20508017 10.1093/intqhc/mzq024

[3] Atalay MO, Aydemir P, Acuner T. The influence of emotional exhaustion on organizational cynicism: the sequential mediating effect of organizational identification and trust in organization. SAGE Open. 2022;12(2):21582440221093343. 10.1177/21582440221093343.

[4] Ayanian JZ, Williams RA. Principles for eliminating racial and ethnic disparities in healthcare. In R. A. Williams, editor, Eliminating healthcare disparities in America. Humana Press; 2007. pp. 377–389. 10.1007/978-1-59745-485-8_18.

[5] Baneshi MR, Talei AR. Dichotomisation of continuous data: Review of methods, advantages, and disadvantages. Int J Cancer Manage. 2011;4(1):e80724. https://brieflands.com/journals/ijcm/articles/80724.

[6] Berwick DM. The moral determinants of health. JAMA. 2020;324(3):225–6. 10.1001/jama.2020.11129. 32530455 10.1001/jama.2020.11129

[7] Birkhäuer J, Gaab J, Kossowsky J, Hasler S, Krummenacher P, Werner C, Gerger H. Trust in the health care professional and health outcome: A meta-analysis. PLoS ONE. 2017;12(2):e0170988. 10.1371/journal.pone.0170988. 28170443 10.1371/journal.pone.0170988 PMC5295692

[8] Bloom DE, Canning D, Kotschy R, Prettner K, Schünemann JJ. Health and economic growth: reconciling the micro and macro evidence (NBER Working Paper No. 26003). National Bureau of Economic Research; 2019. 10.3386/w26003. 10.1016/j.worlddev.2024.106575 PMC10921999 38463754

[9] Buttrick NR, Oishi S. The psychological consequences of income inequality. Soc Pers Psychol Compass. 2017;11(3):e12304. 10.1111/spc3.12304.

[10] Chakroun M. Health and economic growth: new evidence from a panel threshold model. Cogent Econ Finance. 2024;12(1):2331010. 10.1080/23322039.2024.2331010.

[11] Cheung F, Lucas RE. Income inequality is associated with stronger social comparison effects: The effect of relative income on life satisfaction. J Personal Soc Psychol. 2016;110(2):332–41. 10.1037/pspp0000059. 10.1037/pspp0000059 PMC4718872 26191957

[12] Chiu M, Lebenbaum M, Cheng J, de Oliveira C, Kurdyak P. The direct healthcare costs associated with psychological distress and major depression: A population-based cohort study in Ontario, Canada. PLoS ONE. 2017;12(9):e0184268. 10.1371/journal.pone.0184268. 28873469 10.1371/journal.pone.0184268 PMC5584795

[13] Dědeček R, Dudzich V. Exploring the limitations of GDP per capita as an indicator of economic development: A cross-country perspective. Rev Economic Perspect. 2022;22(3):193–217. 10.2478/revecp-2022-0009.

[14] Delhey J, Dragolov G. Why inequality makes Europeans less happy: The role of distrust, status anxiety, and perceived conflict. Eur Sociol Rev. 2014;30(2):151–65. 10.1093/esr/jct033.

[15] Elovainio M, Kivimäki M, Vahtera J. Organizational justice: Evidence of a new psychosocial predictor of health. Am J Public Health. 2002;92(1):105–8. 10.2105/AJPH.92.1.105. 11772771 10.2105/ajph.92.1.105 PMC1447369

[16] Erakhtina AA. Investments in healthcare, life expectancy, and economic growth. Probl Econ Transit. 2022;63(1–3):20–33. 10.1080/10611991.2022.2113312.

[17] Eyer J, Sterling P. Stress-related mortality and social organization. Rev Radical Political Econ. 1977;9(1):1–44. 10.1177/048661347700900103.

[18] Friel S, Marmot MG. Action on the social determinants of health and health inequities goes global. Annu Rev Public Health. 2011;32:225–36. 10.1146/annurev-publhealth-031210-101220. 21219162 10.1146/annurev-publhealth-031210-101220

[19] Ghislandi S, Sanderson WC, Scherbov S. A simple measure of human development: The Human Life Indicator. Popul Dev Rev. 2019;45(1):219–33. 10.1111/padr.12205. 31007310 10.1111/padr.12205 PMC6472489

[20] Grigorian K, Östberg V, Raninen J, Åhlén J, Brolin Låftman S. Prospective associations between psychosomatic complaints in adolescence and depression and anxiety symptoms in young adulthood: A Swedish national cohort study. SSM - Popul Health. 2023;24:101509. 10.1016/j.ssmph.2023.101509. 37720821 10.1016/j.ssmph.2023.101509 PMC10500464

[21] ISSP Research Group. ZA8000 International Social Survey Programme: Health and Health Care II - ISSP 2021. GESIS Data Archive, Cologne. ZA8000 Data file Version 2.0.0; 2024. 10.4232/5.ZA8000.2.0.0.

[22] Jackson B, Kubzansky LD, Wright RJ. Linking perceived unfairness to physical health: The perceived unfairness model. Rev Gen Psychol. 2006;10(1):21–40. 10.1037/1089-2680.10.1.21.

[23] Jokela M, García-Velázquez R, Komulainen K, Savelieva K, Airaksinen J, Gluschkoff K. Specific symptoms of the General Health Questionnaire (GHQ) in predicting persistence of psychological distress: Data from two prospective cohort studies. J Psychiatr Res. 2021;143:550–5. 10.1016/j.jpsychires.2020.11.026. 33243456 10.1016/j.jpsychires.2020.11.026

[24] Kivimäki M, Vahtera J, Elovainio M, Virtanen M, Siegrist J. Effort-reward imbalance, procedural injustice and relational injustice as psychosocial predictors of health: Complementary or redundant models? Occup Environ Med. 2007;64(10):659–65. 10.1136/oem.2006.031310. 17259167 10.1136/oem.2006.031310 PMC2078405

[25] Komlos J. Die US-Variante des Kapitalismus erzeugt gesundheitsgefährdenden chronischen Stress. Wirtschaftsdienst. 2024;104(6):413–9. 10.2478/wd-2024-0108.

[26] Låftman SB, Östberg V, Raninen J. Trust and psychosomatic complaints in adolescence: Findings from a Swedish cohort study. Int J Public Health. 2023;68:1606032. 10.3389/ijph.2023.1606032. 37885767 10.3389/ijph.2023.1606032 PMC10598280

[27] Lange S, Vollmer S. The effect of economic development on population health: A review of the empirical evidence. Br Med Bull. 2017;121(1):47–60. 10.1093/bmb/ldw052. 28069615 10.1093/bmb/ldw052

[28] Lena HF, London B. The political and economic determinants of health outcomes: A cross-national analysis. Int J Health Serv. 1993;23(3):585–602. 10.2190/EQUY-ACG8-X59F-AE99. 8375956 10.2190/EQUY-ACG8-X59F-AE99

[29] Lerch SP, Hänggi R, Bussmann Y, Lörwald A. A model of contributors to a trusting patient-physician relationship: A critical review using a systematic search strategy. BMC Prim Care. 2024;25(1):194. 10.1186/s12875-024-02435-z. 38824511 10.1186/s12875-024-02435-z PMC11143600

[30] Liebig S. Richard Wilkinson and Kate Pickett (2009): The Spirit Level. Why More Equal Societies Almost Always Do Better. Allen Lane Lond Social Justice Res. 2012;25(1):102–7. 10.1007/s11211-012-0148-9.

[31] Livingston V, Jackson-Nevels B, Reddy VV. Social, cultural, and economic determinants of well-being. Encyclopedia. 2022;2(3):1183–99. 10.3390/encyclopedia2030079.

[32] Lu T-P, Rau P-LP, Guo Z, Chen C-L. Factors determining perceptions of fairness in access to hospital outpatient departments in Taiwan. J Health Serv Res Policy. 2018;23(1):15–20. 10.1177/1355819617725546. 29235370 10.1177/1355819617725546

[33] Lucas T. Health consequences and correlates of social justice. In S. B. Gulliver & L. M. Cohen, editors, The Wiley encyclopedia of health psychology. Wiley; 2020. pp. 223–230. 10.1002/9781119057840.ch70.

[34] Luebker M. Inequality, income shares and poverty: the practical meaning of Gini coefficients (TRAVAIL Policy Brief No. 3). International Labour Office; 2010.

[35] MacCallum RC, Zhang S, Preacher KJ, Rucker DD. On the practice of dichotomization of quantitative variables. Psychol Methods. 2002;7(1):19–40. 10.1037/1082-989X.7.1.19. 11928888 10.1037/1082-989x.7.1.19

[36] Mackenbach JP. The persistence of health inequalities in modern welfare states: The explanation of a paradox. Soc Sci Med. 2012;75(4):761–9. 10.1016/j.socscimed.2012.02.031. 22475407 10.1016/j.socscimed.2012.02.031

[37] Marmot M, Wilkinson RG. Psychosocial and material pathways in the relation between income and health: a response to Lynch et al. BMJ. 2001;322(7296):1233–6. 10.1136/bmj.322.7296.1233. 10.1136/bmj.322.7296.1233 PMC1120336 11358781

[38] Missinne S, Meuleman B, Bracke P. The popular legitimacy of European healthcare systems: A multilevel analysis of 24 countries. J Eur Social Policy. 2013;23(3):231–47. 10.1177/0958928713480065.

[39] Mohseni M, Lindstrom M. Social capital, trust in the health-care system and self-rated health: The role of access to health care in a population-based study. Soc Sci Med. 2007;64(7):1373–83. 10.1016/j.socscimed.2006.11.023. 17202025 10.1016/j.socscimed.2006.11.023

[40] Newman DA. Missing data: Five practical guidelines. Organizational Res Methods. 2014;17(4):372–411. 10.1177/1094428114548590.

[41] OECD. Does healthcare deliver? Results from the patient-reported indicator surveys (PaRIS). OECD Publishing; 2025. 10.1787/c8af05a5-en.

[42] Patel V, Burns JK, Dhingra M, Tarver L, Kohrt BA, Lund C. Income inequality and depression: A systematic review and meta-analysis of the association and a scoping review of mechanisms. World Psychiatry. 2018;17(1):76–89. 10.1002/wps.20492. 29352539 10.1002/wps.20492 PMC5775138

[43] Pérez-Arechaederra D, Briones E, Lind A, García-Ortiz L. Perceived organizational justice in care services: Creation and multi-sample validation of a measure. Soc Sci Med. 2014;102:26–32. 10.1016/j.socscimed.2013.11.045. 24565138 10.1016/j.socscimed.2013.11.045

[44] Pratt LA. Serious psychological distress, as measured by the K6, and mortality. Ann Epidemiol. 2009;19(3):202–9. 10.1016/j.annepidem.2008.12.005. 19217003 10.1016/j.annepidem.2008.12.005

[45] Rădoi M, Lupu A. Understanding institutional trust. What does it mean to trust the health system? In: Maturo A, Hošková-Mayerová Š, Soitu DT, Kacprzyk J editors, Studies in Systems, Decision and Control. Vol. 66. Springer International Publishing; 2017. pp. 11–22. 10.1007/978-3-319-40585-8_2.

[46] Radovanovic B. Human development index as a measure of human development. Filozofija i Društvo. 2011;22(3):193–208. 10.2298/FID1103193R.

[47] Rakesh D, Shiba K, Lamont M, Lund C, Pickett KE, VanderWeele TJ, Patel V. Economic inequality and mental health: Causality, mechanisms, and interventions. Ann Rev Clin Psychol. 2025;21(1):353–77. 10.1146/annurev-clinpsy-081423-025710. 40333273 10.1146/annurev-clinpsy-081423-025710

[48] Ridner SH. Psychological distress: Concept analysis. J Adv Nurs. 2004;45(5):536–45. 10.1046/j.1365-2648.2003.02938.x. 15009358 10.1046/j.1365-2648.2003.02938.x

[49] Robbins JM, Ford MT, Tetrick LE. Perceived unfairness and employee health: A meta-analytic integration. J Appl Psychol. 2012;97(2):235–72. 10.1037/a0025408. 21928872 10.1037/a0025408

[50] Schneider SM. Why income inequality is dissatisfying—Perceptions of social status and the inequality-satisfaction link in Europe. Eur Sociol Rev. 2019;35(3):409–30. 10.1093/esr/jcz003.

[51] Shakeri, S., Khodabakhsh Pirkalani, R. Decoding the social mind in depression: a computational dissociation between explicit trust and implicit belief updating. Acta Psychologica. 2026;264:106470. 10.1016/j.actpsy.2026.106470. 10.1016/j.actpsy.2026.106470 41702099

[52] Slavich GM, Roos LG, Mengelkoch S, Webb CA, Shattuck EC, Moriarity DP, Alley JC. Social Safety Theory: Conceptual foundation, underlying mechanisms, and future directions. Health Psychol Rev. 2023;17(1):5–59. 10.1080/17437199.2023.2171900. 36718584 10.1080/17437199.2023.2171900 PMC10161928

[53] Smith CP. First, do no harm: Institutional betrayal and trust in health care organizations. J Multidisciplinary Healthc. 2017;10:133–44. 10.2147/JMDH.S125885. 10.2147/JMDH.S125885 PMC5388348 28435281

[54] Sommet N, Morselli D, Spini D. Income inequality affects the psychological health of only the people facing scarcity. Psychol Sci. 2018;29(12):1911–21. 10.1177/0956797618798620. 10.1177/0956797618798620 30312143

[55] Spiteri J, von Brockdorff P. Economic development and health outcomes: Evidence from cardiovascular disease mortality in Europe. Soc Sci Med. 2019;224:37–44. 10.1016/j.socscimed.2019.01.050. 30738235 10.1016/j.socscimed.2019.01.050

[56] Subramanian SV, Belli P, Kawachi I. The macroeconomic determinants of health. Annu Rev Public Health. 2002;23:287–302. 10.1146/annurev.publhealth.23.100901.140540. 11910064 10.1146/annurev.publhealth.23.100901.140540

[57] Taylor LA, Nong P, Platt J. Fifty years of trust research in health care: A synthetic review. Milbank Q. 2023;101(1):126–78. 10.1111/1468-0009.12598. 36689251 10.1111/1468-0009.12598 PMC10037697

[58] Tibber MS, Walji F, Kirkbride JB, Huddy V. The association between income inequality and adult mental health at the subnational level: A systematic review. Soc Psychiatry Psychiatr Epidemiol. 2022;57(1):1–24. 10.1007/s00127-021-02159-w. 34386869 10.1007/s00127-021-02159-w PMC8761134

[59] United Nations Development Programme. Data Center: Human Development Data. 2026. https://hdr.undp.org/data-center.

[60] United Nations University World Institute for Development. World Income Inequality Database (WIID), Version 6; 2020 May. https://www.wider.unu.edu/node/236613.

[61] van Deurzen I, van Ingen E, van Oorschot WJH. Income inequality and depression: The role of social comparisons and coping resources. Eur Sociol Rev. 2015;31(4):477–89. 10.1093/esr/jcv007.

[62] Wilkinson RG, Pickett KE. Income inequality and social dysfunction. Ann Rev Sociol. 2009;35(1):493–511. 10.1146/annurev-soc-070308-115926.

[63] World Bank Group. Gini index; 2026. https://data.worldbank.org/indicator/SI.POV.GINI.

[64] World Health Organization. Over a billion people living with mental health conditions: services require urgent scale-up. WHO News, 2 September. Geneva: World Health Organization; 2025. https://www.who.int/news/item/02-09-2025-over-a-billion-people-living-with-mental-health-conditions-services-require-urgent-scale-up?.

[65] World Health Organization. World mental health report: Transforming mental health for all. Geneva: World Health Organization; 2022. https://www.who.int/publications/i/item/9789240049338?.

[66] World Health Organization. The world health report 2000: health systems: improving performance. World Health Organ. 2000. https://www.who.int/publications/i/item/924156198X.

[67] Wu D, Lowry PB, Zhang D, Tao Y. Patient trust in physicians matters—Understanding the role of a mobile patient education system and patient-physician communication in improving patient adherence behavior: Field study. J Med Internet Res. 2022;24(12):e42941. 10.2196/42941. 36538351 10.2196/42941 PMC9776535

[68] Yuan Y, Lee KS. General trust in the health care system and general trust in physicians: A multilevel analysis of 30 countries. Int J Comp Sociol. 2022;63(3):91–104. 10.1177/00207152221085571.

[69] Zhu Y, Jha SC, Shutta KH, Huang T, Balasubramanian R, Clish CB, Hankinson SE, Kubzansky LD. Psychological distress and metabolomic markers: A systematic review of posttraumatic stress disorder, anxiety, and subclinical distress. Neurosci Biobehavioral Reviews. 2022;143:104954. 10.1016/j.neubiorev.2022.104954. 10.1016/j.neubiorev.2022.104954 PMC9729460 36368524

