# Supplementary material for: Psychological distress and trust in healthcare: individual perceptions and national contexts - a multilevel analysis
Source: BMC Health Serv Res. 2026 Jun 18;26:852. doi: 10.1186/s12913-026-14948-7 (PMC13282865; doi:10.1186/s12913-026-14948-7)
Supplement: Supplementary file 1 — Supplementary Material 1 [file 12913_2026_14948_MOESM1_ESM.docx]

**Supplementary material**

**Table S1:** *Country-level characteristics: Gini coefficient, Human Development Index (HDI), and average confidence in the healthcare system*

| **Country** | **Gini coefficient** | **HDI (Human Development Index)** | **Average confidence in the healthcare system** |
| --- | --- | --- | --- |
| Australia | 33.8 | 0,954 | 3.46 |
| Austria | 30.7 | 0,930 | 3.54 |
| Croatia | 28.9 | 0,878 | 3.03 |
| Czechia | 26.2 | 0,901 | 3.63 |
| Denmark | 28.3 | 0,958 | 3.71 |
| Finland | 27.7 | 0,949 | 3.90 |
| France | 31.5 | 0,915 | 3.35 |
| Germany | 32.4 | 0,958 | 3.66 |
| Hungary | 29.2 | 0,852 | 3.25 |
| Iceland | 26.8 | 0,967 | 3.21 |
| Israel | 37.9 | 0,918 | 3.24 |
| Italy | 34.8 | 0,908 | 3.10 |
| Japan | 32.3 | 0,922 | 3.19 |
| Netherlands | 25.7 | 0,951 | 3.72 |
| New Zealand | 31.7 | 0,939 | 3.27 |
| Norway | 29.1 | 0,969 | 3.93 |
| Poland | 28.5 | 0,884 | 2.75 |
| Slovakia | 24.1 | 0,859 | 3.09 |
| Slovenia | 24.3 | 0,924 | 3.62 |
| Switzerland | 33.8 | 0,968 | 3.78 |
| United States | 39.7 | 0,921 | 2.94 |
| India* | 32.9 | 0,644 | 3,52 |
| Mexico* | 44.6 | 0,781 | 2,85 |
| Philippines* | 40.7 | 0,710 | 3,38 |
| Russia* | 35.1 | 0,821 | 2,76 |
| South Africa* | 63 | 0,717 | 3,03 |
| Suriname* | 39.2 | 0,690 | 2,62 |
| Thailand* | 34.9 | 0,803 | 2,92 |

*Notes:* *Countries were not included in the analytical sample used in the main manuscript analysis (see Methods section). However, they were retained in the broader country sample used for the robustness check reported in Table S5 of the Supplementary Material. The sources refer to the macro-level indicators described in the Methods section. For the Gini coefficient, data for countries not covered by the World Bank were sourced from the World Income Inequality Database (India, New Zealand, South Africa, Suriname).

**Table S2:** *Logistic multilevel model with 21 countries, dependent variable: psychological distress (ref: Never/ seldom)*

| **Variables** | **OR** | **Lower 95% CI** | **Upper 95% CI** | **p-value** |
| --- | --- | --- | --- | --- |
| *Doctors can be trusted*  *(Ref.: Neither agree nor disagree)* |  |  |  |  |
| Strongly Agree | **0.72** | 0.65 | 0.80 | <.001 |
| Agree | **0.78** | 0.72 | 0.84 | <.001 |
| Disagree/ strongly disagree | **1.14** | 1.01 | 1.30 | 0.030 |
| *Fairness of healthcare (income-based)*  *(Ref.: Neither fair nor unfair)* |  |  |  |  |
| Very fair | 1.11 | 0.96 | 1.28 | 0.169 |
| Somewhat fair | **1.11** | 1.00 | 1.23 | 0.040 |
| Somewhat unfair | **1.18** | 1.08 | 1.28 | <.001 |
| Very unfair | **1.26** | 1.16 | 1.37 | <.001 |
| *Access inequality (income-based)*  *(Ref.: About the same)* |  |  |  |  |
| Much easier (rich) | **1.30** | 1.20 | 1.41 | <.001 |
| Somewhat easier (rich) | **1.09** | 1.00 | 1.19 | 0.048 |
| Somewhat harder/ Much harder (rich) | **1.25** | 1.05 | 1.50 | 0.013 |
| Sex (Ref.: Male) |  |  |  |  |
| Female | **1.47** | 1.39 | 1.56 | <.001 |
| Age | **0.98** | 0.98 | 0.98 | <.001 |
| Education (Ref.: Primary) |  |  |  |  |
| Secondary | **0.82** | 0.69 | 0.97 | 0.024 |
| Tertiary | **0.72** | 0.61 | 0.86 | <.001 |
| Self-rated health (Ref.: Fair/Poor) |  |  |  |  |
| Good | **0.32** | 0.30 | 0.34 | <.001 |
| Very good | **0.15** | 0.14 | 0.16 | <.001 |
| Excellent | **0.08** | 0.07 | 0.09 | <.001 |
| Place of residence (Ref.: Rural area) |  |  |  |  |
| Country village | **1.29** | 1.10 | 1.53 | 0.002 |
| Town / small city | **1.34** | 1.15 | 1.58 | <.001 |
| Suburbs of big city | **1.30** | 1.10 | 1.54 | 0.002 |
| Big city | **1.39** | 1.18 | 1.63 | <.001 |
| *Country-level variables* |  |  |  |  |
| Gini coefficient | **1.26** | 1.10 | 1.44 | <.001 |
| HDI | 1.18 | 0.90 | 1.54 | 0.231 |
| Average confidence in the healthcare  system | 1.09 | 0.89 | 1.33 | 0.375 |

Notes: N = 26 023 individuals nested in 21 countries

**Table S3:** *Multilevel logistic regression model using the extended country sample (28 countries), dependent variable: psychological distress (reference category: never/seldom)*

| **Variables** | **OR** | **Lower 95% CI** | **Upper 95% CI** | **p-value** |
| --- | --- | --- | --- | --- |
| *Doctors can be trusted*  *(Ref.: Neither agree nor disagree)* |  |  |  |  |
| Strongly Agree | **0.71** | 0.65 | 0.77 | <.001 |
| Agree | **0.76** | 0.71 | 0.81 | <.001 |
| Disagree/ strongly disagree | **1.15** | 1.05 | 1.27 | 0.004 |
| *Fairness of healthcare (income-based)*  *(Ref.: Neither fair nor unfair)* |  |  |  |  |
| Very fair | 1.07 | 1.00 | 1.19 | 0.248 |
| Somewhat fair | 0.98 | 0.90 | 1.06 | 0.706 |
| Somewhat unfair | **1.07** | 1.00 | 1.15 | 0.077 |
| Very unfair | **1.20** | 1.12 | 1.29 | <.001 |
| *Access inequality (income-based)*  *(Ref.: About the same)* |  |  |  |  |
| Much easier (rich) | **1.18** | 1.10 | 1.27 | <.001 |
| Somewhat easier (rich) | 1.05 | 0.97 | 1.13 | 0.233 |
| Somewhat harder/ Much harder (rich) | **1.51** | 1.33 | 1.72 | <.001 |
| Sex (Ref.: Male) |  |  |  |  |
| Female | **1.39** | 1.32 | 1.46 | <.001 |
| Age | **0.98** | 0.98 | 0.98 | <.001 |
| Education (Ref.: Primary) |  |  |  |  |
| Secondary | **0.80** | 0.72 | 0.89 | <.001 |
| Tertiary | **0.71** | 0.64 | 0.79 | <.001 |
| Self-rated health (Ref.: Fair/Poor) |  |  |  |  |
| Good | **0.38** | 0.36 | 0.41 | <.001 |
| Very good | **0.19** | 0.18 | 0.20 | <.001 |
| Excellent | **0.14** | 0.13 | 0.16 | <.001 |
| Place of residence (Ref.: Rural area) |  |  |  |  |
| Country village | **1.27** | 1.10 | 1.48 | 0.001 |
| Town / small city | **1.36** | 1.18 | 1.57 | <.001 |
| Suburbs of big city | **1.34** | 1.16 | 1.55 | <.001 |
| Big city | **1.38** | 1.20 | 1.59 | <.001 |
| *Country-level variables* |  |  |  |  |
| Gini coefficient | **1.40** | 1.16 | 1.70 | <.001 |
| HDI | **1.31** | 1.09 | 1.57 | 0.004 |
| Average confidence in the healthcare  system | 1.23 | 1.04 | 1.45 | 0.114 |

Notes: N = 33 783 individuals nested in 28 countries
